# Detection and Identification of Genome Editing in Plants: Challenges and Opportunities

**DOI:** 10.3389/fpls.2019.00236

**Published:** 2019-03-12

**Authors:** Lutz Grohmann, Jens Keilwagen, Nina Duensing, Emilie Dagand, Frank Hartung, Ralf Wilhelm, Joachim Bendiek, Thorben Sprink

**Affiliations:** ^1^ Federal Office of Consumer Protection and Food Safety, Berlin, Germany; ^2^ Institute for Biosafety in Plant Biotechnology, Julius Kühn-Institut, Quedlinburg, Germany

**Keywords:** genome editing, new breeding techniques, GMO, detection, identification, SDN, ODM

## Abstract

Conventional genetic engineering techniques generate modifications in the genome *via* stable integration of DNA elements which do not occur naturally in this combination. Therefore, the resulting organisms and (most) products thereof can unambiguously be identified with event-specific PCR-based methods targeting the insertion site. New breeding techniques such as genome editing diversify the toolbox to generate genetic variability in plants. Several of these techniques can introduce single nucleotide changes without integrating foreign DNA and thereby generate organisms with intended phenotypes. Consequently, such organisms and products thereof might be indistinguishable from naturally occurring or conventionally bred counterparts with established analytical tools. The modifications can entirely resemble random mutations regardless of being spontaneous or induced chemically or *via* irradiation. Therefore, if an identification of these organisms or products thereof is demanded, a new challenge will arise for (official) seed, food, and feed testing laboratories and enforcement institutions. For detailed consideration, we distinguish between the *detection of sequence alterations* – regardless of their origin – the *identification of the process* that generated a specific modification and the *identification of a genotype*, i.e., an organism produced by genome editing carrying a specific genetic alteration in a known background. This article briefly reviews the existing and upcoming detection and identification strategies (including the use of bioinformatics and statistical approaches) in particular for plants developed with genome editing techniques.

## Introduction

For a genetically modified organism (GMO) and the derived food and feed products, the European genetic engineering legislation demands event-specific methods for detection, identification, and quantification before they may be authorized and placed on the market^1^. Market releases of organisms generated through random mutagenesis (resulting from, e.g., irradiation or mutagenic chemicals) do not require analytical methods for post-market identification and traceability, because such organisms are exempt from the obligations of Directive 2001/18/EC on the deliberate release of GMOs. In contrast, organisms developed using genome editing (gene editing) are not exempt, as ruled by the European Court of Justice on July 25th 2018^2^. Consequently, the requirements according to the genetic engineering legislation for detection, identification, and quantification apply for these organisms and food and feed derived thereof. Market releases need to comply with the rigorous legal obligations for risk assessment, labeling, and traceability.

EU-authorized “classic GMOs” are detectable, identifiable, and quantifiable by polymerase chain reaction (PCR) methods, which target the stable integration site of “foreign” DNA elements in a genome, as this is a combination that does not occur naturally. Plants produced by the application of new breeding techniques (NBT) like genome editing, however, may lack integrations of any foreign DNA or corresponding genetic elements commonly used in “classic GMOs.” The application of genome editing aims to minimize the amount of unintended off-target alterations, and subsequent backcrossing and selection steps help to limit the alteration exclusively to the target site without leaving other permanent changes in the genome (e.g., Wang et al., 2014). As a result, the genome sequence of a genome-edited plant may differ only minimally from its parental one (Zhang et al., 2014; Shin et al., 2016).

Genome editing techniques using nucleases can be categorized into site-directed nuclease systems (SDN) 1, 2, and 3 (EFSA, 2012; Podevin et al., 2013). SDN1 applications rely on the endogenous processes of non-homologous end-joining (NHEJ), which is the most common mechanism to repair double-strand DNA breaks in plants. Since NHEJ is an error-prone mechanism, random point mutations frequently occur at the repaired locus (Hsu et al., 2014; Bortesi and Fischer, 2015). Homology-directed repair (HDR) is an alternative repair mechanism, which the cell may apply if a template sequence is available (Sonoda et al., 2006). If this repair template differs by one or a few nucleotides and is otherwise homologous to the autochthonous sequence, the application will be categorized as SDN2 (EFSA, 2012). If longer DNA sequences, which might be of allelic, additional, or foreign origin, are site-specifically integrated into the target genome, this mechanism will be categorized as SDN3 (EFSA, 2012). Oligonucleotide-directed mutagenesis (ODM) does not require the introduction of a nuclease but uses a synthetic single-stranded oligonucleotide, which is complementary to the target sequence, to introduce precise, site-specific modifications of one or a few nucleotides by the cellular mismatch repair mechanism (Mohanta et al., 2017).

As compared to plants generated *via* conventional genetic engineering, the detection of plants obtained by the application of NBTs poses a couple of new challenges. These plants may not contain foreign DNA such as the commonly used cauliflower mosaic virus (CaMV) promoters and terminators (e.g., CaMV P-35S or T-35S). NBTs, including genome editing, offer the possibility to alter the nucleotide sequence specifically. The modifications are often as small as the substitution, insertion, or deletion (indel) of only a single nucleotide.

If genes coding for the genome editing components, e.g., the site-directed nucleases, are stably integrated into the genome of the recipient, the initially regenerated plant will contain foreign DNA. Through subsequent crossing and selection, at best, the locus harboring the integration will be segregated out completely. Then, the offspring used for further breeding will contain the intended genome-edited modification but will not harbor the foreign DNA (null-segregant). Alternatively, genome editing through vector based, transiently expressed nucleases and guide RNA may be applied (Zhang et al., 2016). If transgene-free genome editing is applied by introduction of transcription activator-like effector nuclease (TALEN) proteins or preassembled Cas9 protein-gRNA ribonucleoproteins into cells, no allochthonous DNA will be used and can be expected in the organism at any time (Woo et al., 2015; Metje-Sprink et al., 2018).

German governmental research and regulatory institutions published a scientific report on NBT in plant and animal breeding and their application in the area of nutrition and agriculture^3^. Here, we report the findings concerning detection and identification of genome-edited plants. We focus on whether or not

1. modifications of a plant genome can be detected analytically (detection of a specific sequence);
2. it is analytically possible to prove that a given sequence modification was induced by genome editing or any other specific technique (identification of the process); and
3. a plant generated through genome editing can unambiguously be identified (identification of the genotype).

Evaluating the different methods in this article needs to clarify a main characteristic of plant samples: A sample might be homogeneous, i.e., consisting only of a single genotype, or heterogeneous, i.e., a mixture of various genotypes. *A priori*, it cannot be decided whether a sample taken from a commodity is homogenous or heterogeneous. If it is essential to analyze a homogeneous sample in order to identify a distinct genotype, a single plant has to be tested.

## Analytical Methods for the Detection of Specific Sequences

Various analytical tools are well established and routinely used for “classic” GMO detection. In the following sections, these tools are considered for the applicability for detection of genome-edited plants.

### DNA Amplification-Based Methods

The most common method applied to analyze a locus of interest (e.g., a known genome-edited DNA sequence) is PCR. It requires the knowledge of the target DNA sequence of the modified locus and applies complementary oligonucleotides as primers and a polymerase for cyclic DNA amplification. A large number of standardized reference PCR methods for detection of transgenic constructs and of classical GMOs is available^4^^,^^5^ and might be adapted to genome-edited plants.

If a known insertion is present, PCR-based methods will be state-of-the-art. PCR-based methods are highly specific and sensitive. Based on the experience from GMO testing, it should be feasible to establish event-specific PCR methods targeting larger nucleotide sequence changes induced by genome editing (for example SDN3). Short sequence changes (substitutions or indels of one or a few nucleotides) induced by SDN1, SDN2, or ODM should also be detectable using a specific probe, for example, TaqMan real-time PCR or digital PCR (Stevanato and Biscarini, 2016). Single nucleotide polymorphism (SNP) genotyping approaches can be used to detect very small sequence differences of one or a few nucleotides, provided an adequate reference sequence is available (Huggett et al., 2015; Broccanello et al., 2018). For heterogeneous samples, it was shown that an optimized SNP assay based on digital PCR can detect one mutant within up to 100,000 wild types (Jennings et al., 2014). However, it is questionable whether it will be feasible to develop a robust and specific PCR-based quantification assay for the presence of genome-edited material that is applicable for routine testing of, e.g., composite food samples at the EU-regulative decision levels of 0.9 or 0.1% of genetically modified material (Emons et al., 2018).

### DNA Sequencing-Based Methods

Conventional chain termination (Sanger) sequencing will be suitable for the *targeted* detection of known sequences even if the modifications are small. Especially from homogeneous samples, the altered locus can be amplified and sequenced. It might be unsuitable for heterogeneous samples, but massive parallel sequencing of a specific locus using next generation sequencing (NGS), so-called targeted deep sequencing, is a feasible approach for food and GMO analytics and might be adapted for genome-edited plants (Fraiture et al., 2015; Staats et al., 2016). Efforts and costs for detecting (and quantifying) a known genetic sequence difference can be significantly reduced as compared to whole genome sequencing (WGS).

WGS is increasingly used as an analytical method, including for GMO detection (Wahler et al., 2013; Pauwels et al., 2015; Holst-Jensen et al., 2016). WGS requires no prior information on a specific genetic alteration and can be applied as an *untargeted* detection approach for unknown alterations. NGS platforms can produce millions of small DNA sequence reads in parallel, which need to be processed and compared to some reference using bioinformatics pipelines. Therefore, an adequate reference genome sequence for the respective plant is an indispensable prerequisite for the analysis. The reference genome should be derived from the parental plant, as substantial sequence differences are to be expected even between different lines of the same species, different ecotypes, and between the offspring of one parental plant (Ossowski et al., 2010; Zapata et al., 2016).

Furthermore, the application of WGS is increasingly challenging the larger the genome in question is and the more repetitive sequences are present in the genome. This applies for a variety of crop plants, e.g., the genome of the allohexaploid common wheat (*Triticum aestivum*) (Feldman and Levy, 2012). WGS might find its limitations if applied for the analysis of heterogeneous or contaminated plant samples.

If generated sequence data reveal foreign DNA sequences, it is likely that the genetic modification was introduced intentionally either by genome editing (SDN3) or conventional genetic engineering^6^. However, detected sequences derived from other species need to be carefully evaluated, and their integration into the genome needs to be verified. WGS may generate sequence information not only from the target organism but also from a wide array of contaminants, endophytes or pathogens.

### DNA Hybridization Assays, Protein- and Metabolite-Based Methods

There are a number of alternative analytical approaches (e.g., Southern Blot, DNA Microarrays) that are used to characterize a GMO, but these are of minor relevance for the detection of genome-edited plants (Lusser et al., 2011). DNA hybridization assays generally require a large amount of genetic material and have a comparably low sensitivity. Their specificity also depends on the length of the modification. Therefore, they can only be considered for the (targeted) detection of longer altered nucleotide sequences and/or integrated foreign DNA. From our perspective, they are unsuitable for the detection of small or single nucleotide differences.

Protein-based methods such as immuno-based assays (e.g., ELISA) are applied for “classic” GMO detection (e.g., the transgenic gene product). In addition, mass spectrometry (MS) methods such as MALDI-TOF are available (Lusser et al., 2011). However, alterations detected *via* protein-based approaches need to be confirmed by subsequent DNA analyses.

Metabolite-based methods employing chromatography in combination with mass spectrometry (GC-MS, LC-MS) and nuclear magnetic resonance (NMR) are routinely used for the detection and identification of a broad range of substances. They may allow to detect qualitative differences in a (genome-edited) plant metabolite profile and to identify specific substances, if the analyzed sample is homogeneous, unprocessed, and assuming an appropriate reference is available (Lusser et al., 2011; Frank et al., 2012; Kumar et al., 2017). However, their potential as a detection method is considerably limited because the metabolite pattern is highly dynamic and fluctuating in response to developmental and environmental conditions (Verma and Shukla, 2015). Hence, a detected difference in the metabolite profile is no proof of a genetic modification but merely a hint. Therefore, metabolite-based methods might serve as a tool for screening, e.g., for known metabolites specifically produced through the application of genome editing, but any findings need to be confirmed by subsequent DNA analyses.

## Considerations for the Identification of the Process

After the detection of a specific sequence that is different to the reference, it needs to be clarified whether this sequence occurred naturally or whether it was likely introduced by a genome modification technique. To our knowledge, the application of conventional mutagenesis techniques, such as irradiation or mutagenic chemicals, as well as genome editing applications do not leave specific imprints in the genome. Even for the conventional genetic engineering techniques, it may be impossible to unequivocally identify the specifically applied technique for the integration of foreign DNA, e.g., *Agrobacterium*-mediated or biolistic transfer.

Current analytical strategies allow assessing the similarities between sequence data. They do not allow determining how a sequence alteration was introduced – by genome editing (targeted mutagenesis), classical (untargeted) mutagenesis, or whether it occurred spontaneously. This is in line with the report “New Techniques in Agricultural Biotechnology” of the European Commission’s Scientific Advice Mechanism (SAM, 2017). If the developer describes how an alteration was induced, then it can obviously be linked to the applied technique.

In case the genes coding for the genome editing components are absent, it cannot be deduced from the altered sequence which specific process has been used. For this reason, it cannot be distinguished between conventional genetic engineering and genome editing. We will therefore use the term “genome modification” in the following. However, bioinformatics and statistical considerations might help to evaluate whether a detected sequence was potentially introduced by genome modification.

### Bioinformatics

**Figure 1:**
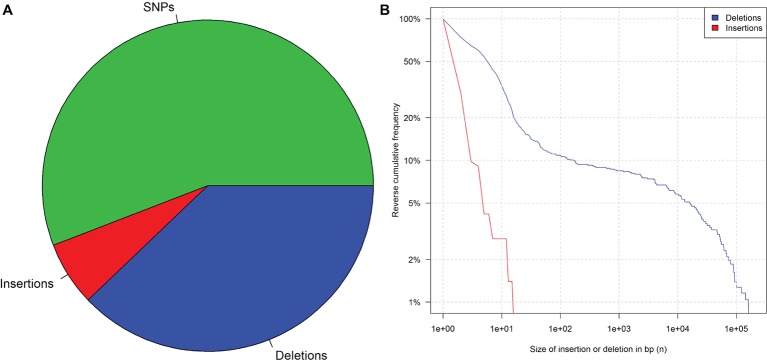
Mutation frequency in 41 rice plants after irradiation and three generations of propagation. **(A)** Pie chart of SNPs, insertions, and deletions. **(B)** Reverse cumulative frequency distribution of indels that are at least n base pairs long. Data from Li et al. (2016), supplementary.

Generally, mutations in genomes of living cells are probably the result of repair mechanisms that are known to be error-prone (Manova and Gruszka, 2015). Many studies have been published to profile the changes that can arise from this natural phenomenon (Salomon and Puchta, 1998; Puchta, 1999; Kirik et al., 2000). Li et al. (2016) published that WGS data of 41 rice plants sequenced a few generations after damaging their DNA with ionizing radiation and their parental plant. An evaluation of these data showed that deletions were more frequent and (on average) larger than insertions (Figure 1). This observation is consistent with what is known about the mechanisms of DNA repair (Puchta, 2005). Insertions larger than 26 bp were not observed, but 15% of the detected deletions were larger than 25 bp. Further studies on rice and *Arabidopsis thaliana* report similar results after induced random mutagenesis (Hirano et al., 2015; Li et al., 2016; Du et al., 2017).

However, considerably longer deletions were observed as well (Figure 1)^7^. In addition, introgression lines harboring chromosomal or segmental substitutions or additions are further examples of long insertions and deletions (Rabinovich, 1998). For this reason, it is impossible to identify the applied technique purely based on the length of a detected indel.

### Statistical Considerations

Lusser et al. (2011) used a simplifying calculation to estimate the minimum length of a unique random sequence in a genome by correlating the genome size with the possible number of combinations for this sequence length. The report of Lusser et al. “assumed that in the case of a plant genome, information on a DNA sequence of at least 20 nucleotides is needed to be in a position to consider a certain DNA sequence as unique and to identify it as the result of a deliberate genetic modification technique.” This estimation exclusively applies to insertions of a sequence of the given length.

In a similar way, the genome sizes of several plant species for the estimation of the length of a sequence which can be statistically considered as unique has been compiled in this paper (Table 1). The probability calculations show that a sequence of 14–17 bp, depending on the genome size of the respective organism, is theoretically expected to be unique. These estimations are based on the simplifying assumption that the four bases are equally distributed and occur statistically independent. However, the complexity of the altered sequence, the amount of repetitive sequences, and the diversity of the genomes within a species are not taken into account.

**Table 1:** Genome sizes of selected (crop) plant species in megabases (1 Mb = 106 bases) (see NCBI, 2018, Sep 6) and the minimal length of a random sequence required to be theoretically unique in a genome of the respective size (simplified assumption purely based on combinatorial possibilities of the four bases within each genome, no other parameters considered).

(Crop) plant species	Haploid genome size (Mb)	Minimum sequence length for theoretical uniqueness in a genome of the respective size (nt)
*Arabidopsis thaliana*	119.67	14
*Oryza sativa*	374.42	15
*Solanum tuberosum*	705.93	15
*Brassica napus*	976.19	15
*Glycine max*	1017.57	15
*Zea mays*	2135.08	16
*Triticum aestivum*	13916.90	17

Only an insertion of a larger sequence, for instance, of a transgene inserted by SDN3, might provide information that can be used for the analyses of its origin. In case a sequence from a different species is detected *via* WGS, it was most likely intentionally introduced into the analyzed genome^6^. If a construct of consecutive foreign genetic elements (e.g., a combination of promoter, coding sequence, and terminator from different species) is identified, it will indicate the application of a genetic modification technique. Search packages like BLAST (Altschul et al., 1990) or k-mer based tools like NIKS (Nordström et al., 2013) can be used to find such DNA sequences within WGS data. Modifications of the foreign DNA, for example, the codon optimization, may impede their identification.

Genome editing techniques can also be applied to introduce targeted mutations of single or a few nucleotides distributed over various loci within one genome (Svitashev et al., 2015; Braatz et al., 2017; Shen et al., 2017). These may be detectable using WGS, but detected alterations need to be evaluated in relation to randomly occurring mutations and considering breeding schemes, i.e., pedigree information and ancestor genotypes.

The expected increase of available genome sequence information in combination with developments and advances in bioinformatics analyses and experience with genome-edited plants will contribute to the improvement of the reliability of these approaches.

## Problems for the Identification of Genotypes

In this section, the question will be discussed, whether the genotype of a genome-edited plant within a plant sample can unambiguously be identified. If a known sequence that is specific for a genetic modification, e.g., a foreign DNA fragment, can be detected in the sample, then the sample will contain a genetically modified genotype that can be identified.

However, most modifications produced by genome editing are very small, down to the substitution, deletion, or insertion of one single nucleotide, which might also occur naturally in non-genome-edited plants (Fauser et al., 2014; Wang et al., 2014; Jacobs et al., 2015). In such cases, the genotype of a modified plant is almost identical to that of the non-modified counterpart, and accurate experimental genotyping is needed to unambiguously identify the genotype. Here, WGS might be considered useful, but it faces a number of substantial problems, e.g.:

1. If the sample is heterogeneous, the identification of a specific genotype will be hampered by the amount and number of other genotypes in the sample. Furthermore, the amount of natural variation in the sample will blur the analysis. If the fragment length of the WGS approach is too short, the linkage between polymorphisms, either naturally occurring or introduced by genome editing, cannot be deduced and the genotypes cannot be determined. Hence, genotyping a heterogeneous sample does not allow identifying individual genotypes in most cases.
2. Avoiding such problems with heterogeneous plant samples, individual plants need to be investigated. For WGS, sufficient amount of DNA is needed. In case of seed samples, a single plant needs to be grown and probed instead of the seed. To avoid missing genotypes, DNA of several plants has to be isolated and sequenced separately, which increases the effort drastically.
3. A high-quality database of all genotypes of genome-edited plants is needed as a reference to unambiguously identify the unique genotype. However, to our knowledge, there is no database providing high-quality genotypes of all genome-edited plants at the present. Furthermore, naturally occurring mutations need to be considered when comparing a sampled sequence to the database. Finally, as mutations and recombination occur naturally during each propagation, there will be low likelihood to trace genotypes that are the offspring of genome-edited plants if the offspring is not included in the database.
4. Sequencing bias of the sequencing technology might lead to an underrepresentation of the genomic region of interest.
5. Due to the small size of the modifications, sequencing errors and other bioinformatics problems increase the potential of false-positive predictions in comparison to conventional GMO analytics.

These problems will be further intensified if the genome of the species is large and/or contains redundant sequences, e.g., in wheat or maize. The amount of time needed and the costs incurred to precisely genotype wheat or other plants with larger genomes seems to render analysis of mixed samples or tests for contaminations infeasible.

## Conclusion

In general, DNA-based procedures are most suitable for the detection of specific sequences in a genome. Without knowledge of the modification, the range of applicable DNA-based methods is limited. PCR requires at least the precise nucleotide sequence information of the locus; thus, PCR cannot be applied if this information is unavailable. Therefore, for the untargeted detection of sequence differences, WGS is currently considered the method of choice, provided an adequate reference genome sequence is available. Once a difference is revealed, this knowledge may be used to develop a targeted (PCR-based) detection method.

Hybridization methods are unsuited to detect very small differences, and the applicability of protein-based and metabolite-based methods for detection is limited. All of them are unsuitable for the routine analysis of commodities.

In contrast to classical genetic engineering, where common or broadly used transgenic elements like typical promoters or terminators combined with a target sequence are used, genome-edited (SDN1 and SDN2, SDN3-based allele exchanges) sites do not carry foreign DNA such as “screening targets,” which makes to our knowledge an untargeted detection of unknown genome-edited loci impossible in most cases. This will challenge market surveillance testing of seeds or food and feed products.

In case a genome sequence difference between two plants was detected, it is challenging to decide whether this difference was introduced using genome editing techniques. Provided that several preconditions apply, bioinformatics and statistical approaches can help to estimate the probability whether genome editing was used. For these considerations, the size and the information encoded in this sequence are essential. For longer insertions, the similarity to DNA of foreign species might be an indicator but can be blurred due to codon optimization. In case of any other differences, additional information as for instance pedigree information in combination with genetic information of the ancestors might help. However, if such information is not available, it will be almost impossible to unambiguously decide on basis of purely statistical approaches, whether or not detected sequence variations were caused by genome editing techniques.

The emergence of further reference genomes or pan-genomes might help to handle some of these problems (Emons et al., 2018). However, using the concept of a pan-genome for the identification of specific genome modification techniques is questionable due to sexual reproduction, introgressions, induced mutagenesis, naturally occurring mutations, and other evolutionary processes. Even with pan-genome information available, to our knowledge, it is not possible to decide for a small difference, e.g., a SNP or a short indel, whether it occurred naturally, whether it was introduced by mutagenesis using chemicals or radiation, or whether it was introduced by genome editing.

The genotype of a plant from a homogeneous sample might be identified in specific cases, e.g., in the presence of specific sequences. However, it will be much harder for most practical cases. As mentioned above, the identification of specific genotypes in heterogeneous samples (commodities) demands a number of essential prerequisites which are commonly not given. However, if the prerequisites are met, the analyses will be very expensive and time consuming. All these considerations are based on an appropriate documentation, e.g., origin and pedigree, of the samples that have to be analyzed. Unambiguous detection of hidden admixtures will still be impossible.

## Data Availability

Publicly available datasets were analyzed in this study. These data can be found here: https://www.cell.com/cms/10.1016/j.molp.2016.03.009/attachment/ecde2a05-a059-4556-90bd-e1dd7650f003/mmc2.xls.

## Author Contributions

LG and JK equally explored the core of the topic with regard to detection and bioinformatics methods and prepared the manuscript. All authors contributed equally in the discussion and conclusions, reviewed, read, and approved the manuscript.

### Disclaimer

The views or positions expressed in this publication do not necessarily represent in legal terms the official position of the institutions or organization the authors work for.

### Conflict of Interest Statement

The authors declare that the research was conducted in the absence of any commercial or financial relationships that could be construed as a potential conflict of interest.

## Footnotes

^1^Directive 2001/18/EC and Regulation (EC) No 1829/2003.

^2^https://curia.europa.eu/jcms/jcms/p1_1217550/en/

^3^https://www.bvl.bund.de/DE/06_Gentechnik/02_Verbraucher/Bericht_Neue_Zuechtungstechniken/gentechnik_Neue_Zuechtungstechniken_node.html;jsessionid=B1C3F310A72AB446ADAE015D0AC88497.1_cid350

^4^http://gmo-crl.jrc.ec.europa.eu/gmomethods/

^5^http://www.euginius.eu/euginius/pages/home.jsf

^6^The integration of nucleic acid sequences of foreign organisms can, albeit very rarely, also occur naturally, as seen in the sweet potato, which was shown to contain *Agrobacterium* genes (Kyndt et al., 2015).

^7^It should be kept in mind that the publicly available data analyzed here were produced by bioinformatics tools that are not expected to report long structural variants (i.e., 50 bases or more as defined by the Structural Variation Analysis Group).

## References

[ref1] Altschul S. F. Gish W. Miller W. Myers E. W. Lipman D. J. (1990). Basic local alignment search tool. J. Mol. Biol. 215, 403–410. 10.1016/S0022-2836(05)80360-2, PMID: 2231712

[ref2] Bortesi L. Fischer R. (2015). The CRISPR/Cas9 system for plant genome editing and beyond. Biotechnol. Adv. 33, 41–52. 10.1016/j.biotechadv.2014.12.006, PMID: 25536441

[ref3] Braatz J. Harloff H. J. Mascher M. Stein N. Himmelbach A. Jung C. (2017). CRISPR-Cas9 targeted mutagenesis leads to simultaneous modification of different homoeologous gene copies in polyploid oilseed rape (*Brassica napus*). Plant Physiol. 174, 935–942. 10.1104/pp.17.00426, PMID: 28584067 PMC5462057

[ref4] Broccanello C. Chiodi C. Funk A. McGrath J. M. Panella L. Stevanato P. (2018). Comparison of three PCR-based assays for SNP genotyping in plants. Plant Methods 14:28. 10.1186/s13007-018-0295-6 29610576 PMC5872507

[ref5] Du Y. Luo S. Li X. Yang J. Cui T. Li W. (2017). Identification of substitutions and small insertion-deletions induced by carbon-ion beam irradiation in *Arabidopsis thaliana*. Front. Plant Sci. 8:1851. 10.3389/fpls.2017.01851 29163581 PMC5665000

[ref6] EFSA (2012). Scientific opinion addressing the safety assessment of plants developed using zinc finger nuclease 3 and other site-directed nucleases with similar function. EFSA J. 10:2943. 10.2903/j.efsa.2012.2943

[ref7] Emons H. Broothaerts W. Bonfini L. Corbisier P. Gatto F. Jacchia S. (2018). Challenges for the detection of genetically modified food or feed originating from genome editing, EUR 29391 EN, Publications Office of the European Union, Luxembourg, 2018. 10.2760/732526

[ref8] Fauser F. Schiml S. Puchta H. (2014). Both CRISPR/Cas-based nucleases and nickases can be used efficiently for genome engineering in *Arabidopsis thaliana*. Plant J. 79, 348–359. 10.1111/tpj.12554, PMID: 24836556

[ref9] Feldman M. Levy A. A. (2012). Genome evolution due to allopolyploidization in wheat. Genetics 192, 763–774. 10.1534/genetics.112.146316, PMID: 23135324 PMC3522158

[ref10] Fraiture M. A. Herman P. Taverniers I. De Loose M. Deforce D. Roosens N. H. (2015). Current and new approaches in gmo detection: challenges and solutions. Biomed. Res. Int. 2015:392872. 10.1155/2015/392872, PMID: 26550567 PMC4624882

[ref11] Frank T. Röhlig R. M. Davies H. V. Barros E. Engel K. H. (2012). Metabolite profiling of maize kernels-genetic modification versus environmental influence. J. Agric. Food Chem. 60, 3005–3012. 10.1021/jf204167t, PMID: 22375597

[ref12] Hirano T. Kazama Y. Ishii K. Ohbu S. Shirakawa Y. Abe T. (2015). Comprehensive identification of mutations induced by heavy-ion beam irradiation in *Arabidopsis thaliana*. Plant J. 82, 93–104. 10.1111/tpj.12793, PMID: 25690092

[ref13] Holst-Jensen A. Spilsberg B. Arulandhu A. J. Kok E. Shi J. Zel J. (2016). Application of whole genome shotgun sequencing for detection and characterization of genetically modified organisms and derived products. Anal. Bioanal. Chem. 408, 4595–4614. 10.1007/s00216-016-9549-1, PMID: 27100228 PMC4909802

[ref14] Hsu P. D. Lander E. S. Zhang F. (2014). Development and applications of CRISPR-Cas9 for genome engineering. Cell 157, 1262–1278. 10.1016/j.cell.2014.05.010, PMID: 24906146 PMC4343198

[ref15] Huggett J. F. Cowen S. Foy C. A. (2015). Considerations for digital PCR as an accurate molecular diagnostic tool. Clin. Chem. 6, 79–88. 10.1373/clinchem.2014.221366 25338683

[ref16] Jacobs T. B. LaFayette P. R. Schmitz R. J. Parrott W. A. (2015). Targeted genome modifications in soybean with CRISPR/Cas9. BMC Biotechnol. 15:16. 10.1186/s12896-015-0131-2 25879861 PMC4365529

[ref17] Jennings L. J. George D. Czech J. Yu M. Joseph L. (2014). Detection and quantification of BCR-ABL1 fusion transcripts by droplet digital PCR. J. Mol. Diagn. 16, 174–179. 10.1016/j.jmoldx.2013.10.007, PMID: 24389534

[ref18] Kirik A. Salomon S. Puchta H. (2000). Species-specific double-strand break repair and genome evolution in plants. EMBO J. 19, 5562–5566. 10.1093/emboj/19.20.5562, PMID: 11032823 PMC314016

[ref19] Kumar A. Mosa K. A. Ji L. Kage U. Dhokane D. Karre S. (2017). Metabolomics assisted biotechnological interventions for developing plant-based functional foods and nutraceuticals. Crit. Rev. Food Sci. Nutr. 58, 1791–1807. 10.1080/10408398.2017.1285752 28272908

[ref20] Kyndt T. Quispe D. Zhai H. Jarret R. Ghislain M. Liu Q. (2015). The genome of cultivated sweet potato contains *Agrobacterium* T-DNAs with expressed genes: an example of a naturally transgenic food crop. Proc. Natl. Acad. Sci. U.S.A. 112, 5844–5849. 10.1073/pnas.1419685112 25902487 PMC4426443

[ref21] Li G. Chern M. Jain R. Martin J. A. Schackwitz W. S. Jiang L.. (2016). Genome-wide sequencing of 41 rice (*Oryza sativa* L.) mutated lines reveals diverse mutations induced by fast-neutron irradiation. Mol. Plant 9, 1078–1081. 10.1016/j.molp.2016.03.009, PMID: 27018389

[ref22] Lusser M. Parisi C. Plan D. Rodríguez-Cerezo E. (2011). New plant breeding techniques. State-of-the-art and prospects for commercial development. (Brussels: Joint Research Centre Technical Report EUR 24760. European Commission Joint Research Centre). ISBN 978-92-79-19715-4. ISSN 1018-5593.

[ref23] Manova V. Gruszka D. (2015). DNA damage and repair in plants—from models to crops. Front. Plant Sci. 6:885. 10.3389/fpls.2015.00885, PMID: 26557130 PMC4617055

[ref24] Metje-Sprink J. Menz J. Modrzejewski D. Sprink T. (2018). DNA-free genome editing: past, present and future. Front. Plant Sci. 9:1957. 10.3389/fpls.2018.01957, PMID: 30693009 PMC6339908

[ref25] Mohanta T. K. Bashir T. Hashem A. Abd_Allah E. F. Bae H. (2017). Genome editing tools in plants. Genes 8:399. 10.3390/genes8120399, PMID: 29257124 PMC5748717

[ref26] NCBI (2018). National Center for Biotechnology Information-Genome Information. https://www.ncbi.nlm.nih.gov/genome/browse/#!/overview/.

[ref27] Nordström K. J. Albani M. C. James G. V. Gutjahr C. Hartwig B. Turck F.. (2013). Mutation identification by direct comparison of whole-genome sequencing data from mutant and wild-type individuals using k-mers. Nat. Biotechnol. 31, 325–330. 10.1038/nbt.2515, PMID: 23475072

[ref28] Ossowski S. Schneeberger K. Lucas-Lledó J. I. Warthmann N. Clark R. M. Shaw R. G. (2010). The rate and molecular spectrum of spontaneous mutations in *Arabidopsis thaliana*. Science 327, 92–94. 10.1126/science.1180677 20044577 PMC3878865

[ref29] Pauwels K. De Keersmaecker S. De Schrijver A. du Jardin P. Roosens N. Herman P. (2015). Next-generation sequencing as a tool for the molecular characterisation and risk assessment of genetically modified plants: added value or not? Trends Food Sci. Technol. 45, 319–326. 10.1016/j.tifs.2015.07.009

[ref30] Podevin N. Davies H. V. Hartung F. Nogué F. Casacuberta J. M. (2013). Site-directed nucleases: a paradigm shift in predictable, knowledge-based plant breeding. Trends Biotechnol. 31, 375–383. 10.1016/j.tibtech.2013.03.004, PMID: 23601269

[ref31] Puchta H. (1999). Double-strand break-induced recombination between ectopic homologous sequences in somatic plant cells. Genetics 152, 1173–1181. PMID: 10388832 10.1093/genetics/152.3.1173 PMC1460648

[ref32] Puchta H. (2005). The repair of double-strand breaks in plants: mechanisms and consequences for genome evolution. J. Exp. Bot. 56, 1–14. 10.1093/jxb/eri025, PMID: 15557293

[ref33] Rabinovich S. V. (1998). Importance of wheat-rye translocations for breeding modern cultivar of *Triticum aestivum* L. Euphytica 100, 323–340. 10.1023/A:1018361819215

[ref34] Salomon S. Puchta H. (1998). Capture of genomic and T-DNA sequences during double-strand break repair in somatic plant cells. EMBO J. 17, 6086–6095. 10.1093/emboj/17.20.6086, PMID: 9774352 PMC1170935

[ref35] SAM (2017). Independent scientific advice for policymaking. New Techniques in Agricultural Biotechnology, High Level Group of Scientific Advisors. Explanatory Note 02. Scientific Advice Mechanism.

[ref36] Shen L. Hua Y. Fu Y. Li J. Liu Q. Jiao X.. (2017). Rapid generation of genetic diversity by multiplex CRISPR/Cas9 genome editing in rice. Sci. China Life Sci. 60, 506–515. 10.1007/s11427-017-9008-8, PMID: 28349304

[ref37] Shin S. E. Lim J. M. Koh H. G. Kim E. K. Kang N. K. Jeon S. (2016). CRISPR/Cas9-induced knockout and knock-in mutations in *Chlamydomonas reinhardtii*. Sci. Rep. 6:27810. 10.1038/srep27810 27291619 PMC4904240

[ref38] Sonoda E. Hochegger H. Saberi A. Taniguchi Y. Takeda S. (2006). Differential usage of non-homologous end-joining and homologous recombination in double strand break repair. DNA Repair 5, 1021–1029. 10.1016/j.dnarep.2006.05.022, PMID: 16807135

[ref39] Staats M. Arulandhu A. J. Gravendeel B. Holst-Jensen A. Scholtens I. Peelen T.. (2016). Advances in DNA metabarcoding for food and wildlife forensic species identification. Anal. Bioanal. Chem. 408, 4615–4630. 10.1007/s00216-016-9595-8, PMID: 27178552 PMC4909793

[ref40] Stevanato P. Biscarini F. (2016). Digital PCR as new approach to SNP genotyping in sugar beet. Sugar Tech. 18, 429–432. 10.1007/s12355-015-0408-8

[ref41] Svitashev S. Young J. K. Schwartz C. Gao H. Falco S. C. Cigan A. M. (2015). Targeted mutagenesis, precise gene editing, and site-specific gene insertion in maize using Cas9 and guide RNA. Plant Physiol. 169, 931–945. 10.1104/pp.15.00793, PMID: 26269544 PMC4587463

[ref42] Verma B. Shukla S. (2015). Impact of various factors responsible for fluctuation in plant secondary metabolites. J. Appl. Res. Med. Aromat. Plants 2, 105–113. 10.1016/j.jarmap.2015.09.002

[ref43] Wahler D. Schauser L. Bendiek J. Grohmann L. (2013). Next-generation sequencing as a tool for detailed molecular characterisation of genomic insertions and flanking regions in genetically modified plants: a pilot study using a rice event unauthorised in the EU. Food Anal. Methods 6, 1718–1727. 10.1007/s12161-013-9673-x

[ref44] Wang Y. Cheng X. Shan Q. Zhang Y. Liu J. Gao C.. (2014). Simultaneous editing of three homoeoalleles in hexaploid bread wheat confers heritable resistance to powdery mildew. Nat. Biotechnol. 32, 947–951. 10.1038/nbt.2969, PMID: 25038773

[ref45] Woo J. W. Kim J. Kwon S. I. Corvalán C. Cho C. W. Kim H.. (2015). DNA-free genome editing in plants with preassembled CRISPR-Cas9 ribonucleoproteins. Nat. Biotechnol. 33, 1162–1164. 10.1038/nbt.3389, PMID: 26479191

[ref46] Zapata L. Ding J. Willing E. M. Hartwig B. Bezdan D. Jiao W. B. (2016). Chromosome-level assembly of *Arabidopsis thaliana* Ler reveals the extent of translocation and inversion polymorphisms. Proc. Natl. Acad. Sci. U.S.A. 113, 4052–4060. 10.1073/pnas.1607532113 PMC4948326 27354520

[ref47] Zhang Y. Liang Z. Zong Y. Wang Y. Liu J. Chen K. (2016). Efficient and transgene-free genome editing in wheat through transient expression of CRISPR/Cas9 DNA or RNA. Nat. Commun. 7:12617. 10.1038/ncomms12617 27558837 PMC5007326

[ref48] Zhang H. Zhang J. Wei P. Zhang B. Gou F. Feng Z.. (2014). The CRISPR/Cas9 system produces specific and homozygous targeted gene editing in rice in one generation. Plant Biotechnol. J. 12, 797–807. 10.1111/pbi.12200, PMID: 24854982

